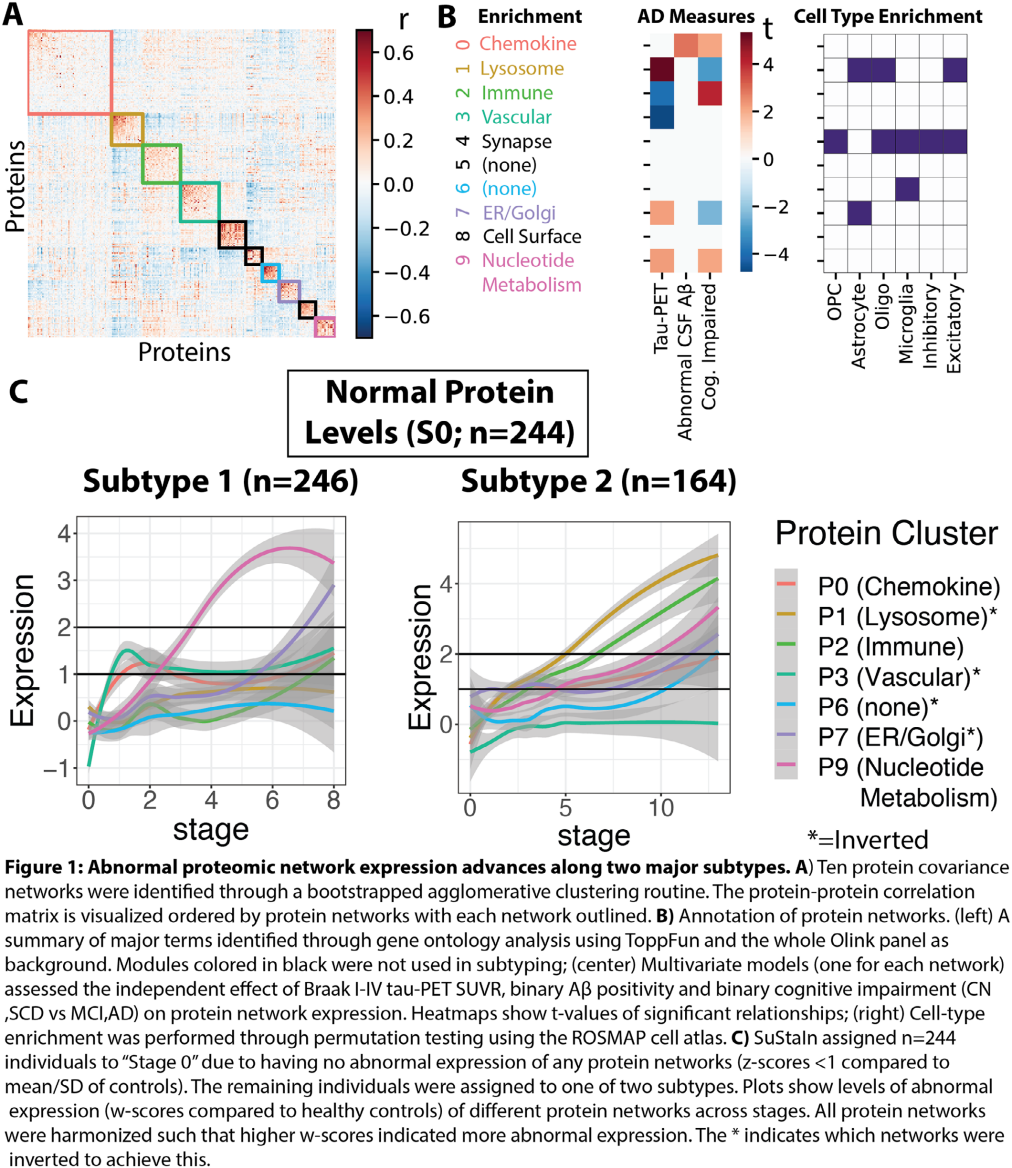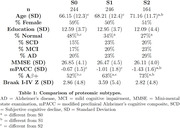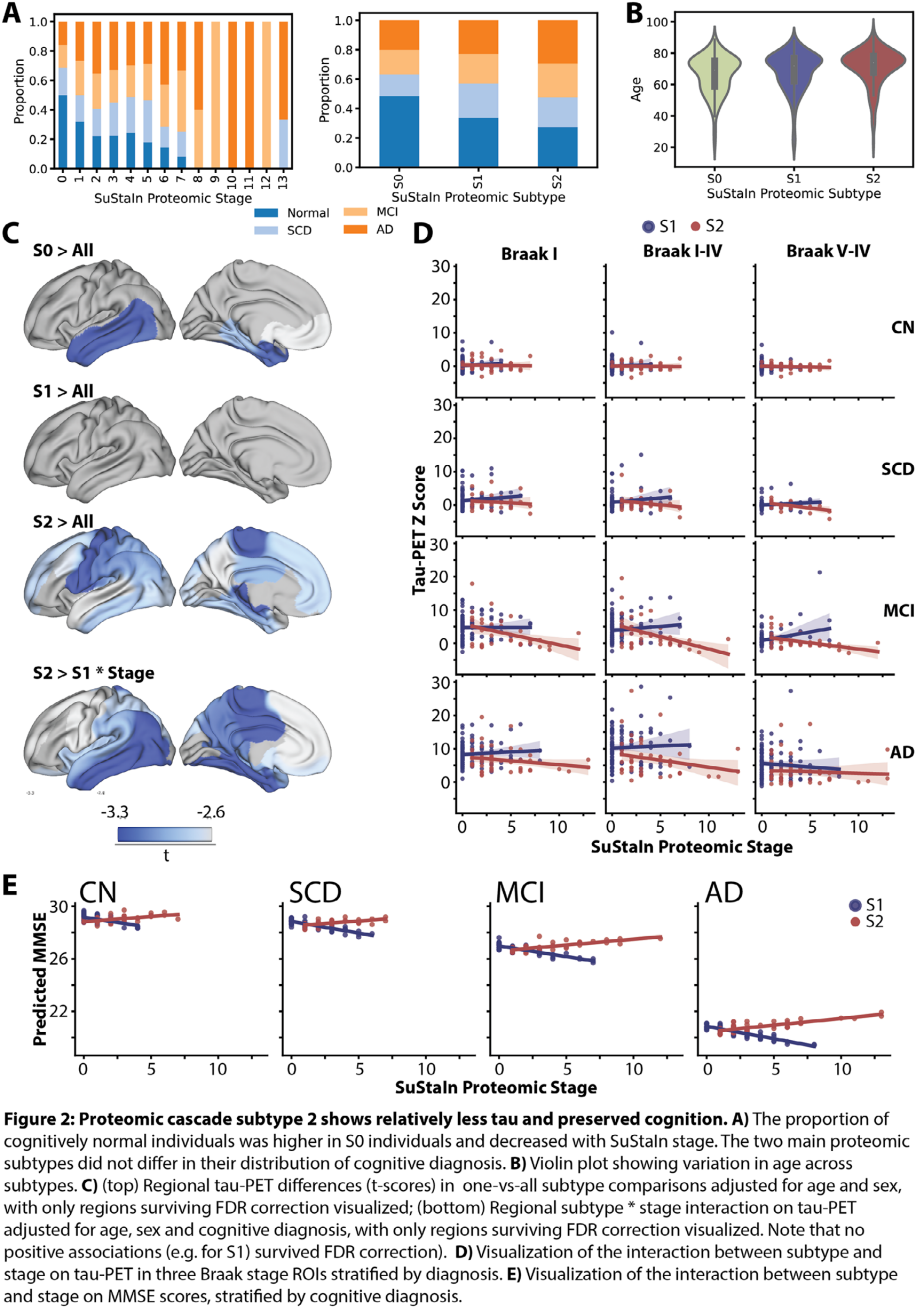# Individual variation in proteomic cascades along the Alzheimer’s disease spectrum

**DOI:** 10.1002/alz.091914

**Published:** 2025-01-09

**Authors:** Jacob W. Vogel, Gabriele Vilkaite, Alexa Pichet Binette, Ines Hristovska, Yu Xiao, Sophie E. Mastenbroek, Jonathan Rittmo, Shorena Janelidze, Sebastian Palmqvist, Gemma Salvadó, Ruben Smith, Erik Stomrud, Olof Strandberg, Alexandra L. Young, Rik Ossenkoppele, Niklas Mattsson‐Carlgren, Oskar Hansson

**Affiliations:** ^1^ Department of Clinical Sciences Malmö, SciLifeLab, Lund University, Lund Sweden; ^2^ Clinical Memory Research Unit, Department of Clinical Sciences, Lund University, Lund Sweden; ^3^ Memory Clinic, Skåne University Hospital, Malmö Sweden; ^4^ Clinical Memory Research Unit, Lund University, Lund Sweden; ^5^ Clinical Memory Research Unit, Department of Clinical Sciences Malmö, Faculty of Medicine, Lund University, Lund Sweden; ^6^ Centre for Medical Image Computing, Department of Computer Science, University College London, London UK; ^7^ Amsterdam Neuroscience, Neurodegeneration, Vrije Universiteit Amsterdam, Amsterdam Netherlands; ^8^ Alzheimer Center Amsterdam, Neurology, Vrije Universiteit Amsterdam, Amsterdam UMC location VUmc, Amsterdam Netherlands

## Abstract

**Background:**

Cerebrospinal fluid (CSF) proteomics allows for characterization of multiple disease‐related biological processes *in vivo*. These processes likely occur along temporal cascades mirroring disease evolution. This study describes interindividual variation in these cascades, in the context of Alzheimer’s disease.

**Method:**

Participants were recruited from the Swedish BioFINDER‐2 study, diagnosed as cognitively normal (n=251; CN), subjective cognitive decline (SCD; n=129), mild cognitive impairment (MCI; n=134) or Alzheimer’s dementia (AD; n=159). All MCI and AD were β‐amyloid (Aβ)‐positive via CSF Aβ42/40 ratio. CSF was sequenced with the Olink Explore 3072 panel, resulting in 1331 proteins after quality control. All participants also underwent [18F]RO948 tau‐PET scans. Age, sex, and the first non‐disease‐related principal components (n=3) were regressed from the proteomic data. A bootstrapped hierarchical clustering approach extracted and annotated ten networks of co‐expressed proteins (Figure 1A). Networks with univariate relationships to Aβ, tau or diagnosis (n=7) were submitted to the Subtype and Stage Inference (SuStaIn) algorithm (Figure 1B). SuStaIn assigned participants to a subtype and stage based on sequential abnormality of proteomic network expression. Regression models assessed SubtypeXStage interactions on regional tau‐PET or MMSE, adjusting for age, sex and diagnosis.

**Result:**

A two‐subtype model best fit the data (Table 1). N=244 showed normal protein network levels (S0). Subtype‐1 (S1) showed early and sustained abnormality in vascular (P3) and chemokine (P0) networks, followed by increasing abnormality of a nucleotide‐metabolism network (P9). Subtype‐2 (S2) showed increasing abnormality in lysosomal (P1) and immune (P2) networks (Figure 1C). CN were more likely to be S0 (𝛘2=23.2, p<0.001) and a lower stage (F=20.5, p<0.001), but S1 and S2 did not differ by diagnosis (Figure 2A). S0 and S2 showed decreased tau‐PET load compared to other groups (Figure 2C). Subtype*stage interactions showed S2 participants had less tau‐PET load and higher MMSE scores at more advanced stages compared to S1 (Figure 2C‐E).

**Conclusion:**

Two proteomic cascades emerged along the AD continuum, with one accompanying relatively lower tau levels and preserved cognition. Longitudinal follow‐up will reveal if this cascade is indicative of increased vulnerability or resilience of AD pathology.